# Functional connectivity in resting-state fMRI (rs-fMRI) in opioid use disorder

**DOI:** 10.1140/epjs/s11734-025-01591-2

**Published:** 2025-03-25

**Authors:** Neli Atanasova, Anna Todeva-Radneva, Kristina Stoyanova, Elena Psederska, Drozdstoy Stoyanov, Nikoleta Traykova, Jasmin Vassileva

**Affiliations:** 1 Strategic Research and Innovation Program for the Development of MU-PLOVDIV–(SRIPD-MUP), European Union-NextGeneration EU, BG-RRP-2.004-0007-C01, Plovdiv, Bulgaria; 2 Research Institute at Medical University of Plovdiv, Medical University of Plovdiv, 15A Vasil Aprilov Blvd., Plovdiv, 4002 Bulgaria; 3 Department of Psychiatry and Medical Psychology, Medical University Plovdiv, 15A Vasil Aprilov Blvd., Plovdiv, 4002 Bulgaria; 4 Department of Cognitive Science and Psychology, New Bulgarian University, 21 Montevideo Str., Sofia, 1618 Bulgaria; 5 Institute for Drug and Alcohol Studies, Virginia Commonwealth University, 203 E. Cary Street, Richmond, VA 23219 USA; 6 Department of Psychiatry, 501 N. 2nd Street, Richmond, VA 23219 USA

## Abstract

This mini-review examines functional connectivity in resting-state functional magnetic resonance imaging (rs-fMRI) among opioid users. The goal is to summarize existing research data and clarify the implications of altered brain connectivity in this population. The first part of the review addresses the critical question of how opioid addiction influences the functional connectivity of key brain networks, such as the default mode network (DMN), salience network (SN), and executive control network (ECN). It examines the neurological basis of opioid addiction, the principles of rs-fMRI, different methodologies employed in this type of research, and inconsistencies and methodological challenges that complicate the interpretation of findings. The second part of the article presents findings derived from our ongoing research in the field. We tested 42 participants of whom 23 healthy controls and 19 patients with opioid use disorder. Each participant underwent an MRI scanning procedure comprised of structural, resting-state and task sequences. The neuroimaging data was processed using the CONN Toolbox running on MATLAB. Our preliminary rs-fMRI findings reveal significant disruptions in functional connectivity in individuals with opioid addiction within DMN and SN networks involved in cognitive functions such as decision-making and impulse control. The review concludes by emphasizing the importance of standardizing research practices, conducting longitudinal randomized studies, and developing a more holistic approach to understanding the effects of heroin addiction. These efforts would contribute to the development of personalized and effective intervention strategies.

**Supplementary Information:**

The online version contains supplementary material available at 10.1140/epjs/s11734-025-01591-2.

## Introduction

Opioid use continues to pose significant global health challenges due to its severe psychological and physiological consequences. Research indicates that chronic opioid use is associated with long-lasting structural and functional alterations in the central nervous system (CNS) and concomitant cognitive (e.g. decision-making) and behavioral (e.g. disinhibition) impairments [1]. Nevertheless, the precise pathophysiological mechanisms of opioid use disorder (OUD) remain elusive, which hinders accurate diagnosis and prognosis and the development of effective prevention and intervention strategies. In this context, we suggest that utilizing non-invasive neuroimaging methods such as resting-state functional magnetic resonance imaging (rs-fMRI) enables researchers to explore complex patterns of functional connectivity across various brain networks in OUD, which may facilitate advancements in existing preventative, therapeutic, and prognostic approaches.

This mini-review aims to explore and summarize current findings in the field, while also sharing insights derived from our ongoing research. In addition, we aim to create a synthesis of the diverse body of literature employing rs-fMRI to provide additional evidence about the specific OUD-related brain alterations that may improve our understanding of the pathophysiology of opioid addiction. The focus of this mini-review will be the inter- and intranetwork aberrations in opioid users of the Default Mode Network (DMN), Salience Network (SN), and Executive Control Network (ECN), implicated in addiction [2, 3].

## Impact of opioid addiction on the default mode, salience, and executive control networks

Functional magnetic resonance imaging (fMRI), particularly resting-state fMRI (rs-fMRI), is a key method for studying brain connectivity, especially in uncooperative patients or those with cognitive impairments [4]. Rs-fMRI uses the blood oxygen-level-dependent (BOLD) signal to detect spontaneous brain activity, reflecting changes in oxy- and deoxyhemoglobin levels due to local blood flow increases during activation [5]. This reveals resting-state networks (RSNs) like the ECN, SN, and DMN, which are critical for understanding normal and abnormal brain function [6].

Data processing relies on platforms such as MATLAB [7], AFNI [8], and FSL [9]. Common analyses include Seed-Based Connectivity (SBC) for hypothesis-driven studies [10] and Independent Component Analysis (ICA) for identifying large-scale networks [11]. However, challenges such as variability from aging, psychiatric conditions, or physiological noise [6], along with replicability issues and the need for multidisciplinary expertise, complicate interpretation [12]. Despite these limitations, rs-fMRI offers significant potential for advancing our understanding of brain connectivity in health and disease [4].

Prior studies exploring the impact of opioid addiction on brain connectivity have emphasized significant alterations in aforementioned key brain networks implicated in addiction [2]. Chronic opioid use affects these networks, contributing to severe cognitive and behavioral deficits often observed among affected individuals.

These networks are implicated in the Addictions Neuroclinical Assessment (ANA) framework [3], an influential neuroscience-based framework for understanding addictive disorders, which focuses on three neurofunctional domains commonly affected by addiction: Executive Function (EF), Incentive Salience (IS), and Negative Emotionality (NE). These domains map onto the three stages of addiction: binge-intoxication, withdrawal-negative affect, and preoccupation-anticipation. Heightened IS drives the intense cravings and reward-seeking behaviors characteristic of the binge-intoxication stage. Impaired EF contributes to the loss of control and compulsive drug seeking seen in the preoccupation-anticipation stage. Finally, increased NA, including stress and anxiety, fuels the withdrawal-negative affect stage, motivating continued drug use to alleviate these negative feelings.

Research reveals that the DMN, typically active during rest and associated with self-referential thinking and mind-wandering, shows altered connectivity in opioid users. For example, Liu and colleagues [13], using graph theory analysis, demonstrated abnormal topological properties in the DMN in abstinent heroin users, revealing increased functional connectivity in the medial frontal gyrus (meFG) and decreased connectivity in the anterior cingulate cortex (ACC). OUD-related alterations in this network were also supported by a study by Li et al. [14], which utilized group ICA analysis and identified two main DMN subnetworks: anterior and posterior. The study found decreased functional connectivity in opioid users in the anterior DMN subnetwork, specifically in the dorsal medial prefrontal cortex. Moreover, the abnormal functional connectivity within the anterior subnetwork of the DMN was associated with drug craving severity, which further illustrates the crucial role of the DMN in opioid addiction.

The SN governs the dynamic interactions between brain networks to prioritize the processing of salient stimuli. The central brain regions involved in the SN include the anterior insula, anterior and dorsal cingulate cortex, amygdala, and the supramarginal gyrus of the parietal cortex [15]. Prior studies provide evidence that opioid addiction significantly affects the normal functioning of the SN. For example, Sun et al. [16] identified extensive disruptions in structural connectivity within attention systems, including the SN in opioid users. In addition, they observed that higher connectivity strength correlated with increased impulsivity and daily heroin dose in their sample. Similar functional changes were further observed in a resting-state EEG study [17], which found reduced connectivity in parietal regions, along with abnormally strong connections in left occipital regions among abstinent heroin users. Similarly, Jin et al. [18] conducted an integrated resting-state PET/fMRI study, reporting both reduced glucose metabolism and reduced metabolic connectivity within the SN, specifically in the anterior insula, also observed by Zhang et al. [19], and in the inferior parietal lobe, suggesting impairments in the network’s cognitive control functions among heroin users.

The ECN, vital for high-level cognitive processes such as decision-making and executive functions, also exhibits impaired connectivity in opioid users. Qiu et al. [20] reported significantly lower regional homogeneity in the medial orbitofrontal cortex (OFC) and other regions associated with decision-making in opioid users. Jiang et al. [21] further illustrate ECN disruption in OUD, reporting that heroin users were characterized by reduced nodal centrality in cognitive control areas such as the middle cingulate gyrus and middle frontal gyrus, indicating a compromised ability of the ECN to effectively regulate cognitive processes. These ECN functional connectivity impairments are in line with neurocognitive studies revealing notable deficits in decision-making in opioid users that are observable even after protracted abstinence from opioid use [22–24].

Broad abnormalities in measures of functional connectivity further support these network-specific disruptions in OUD. For example, Pandria et al. [25] reviewed findings from prior studies that uncovered unusual network topologies in opioid users, characterized by stronger connectivity between the nucleus accumbens and both ventral and rostral aspects of the anterior cingulate cortex and medial orbitofrontal cortex, and weaker connectivity between the medial-dorsolateral prefrontal cortex and the lateral OFC, as well as the dorsal ACC. Structural deficits have also been identified among individuals with OUD, such as reduced gray matter volume in areas critical for cognitive functions and decision-making. These findings align with those of Yuan et al. [26], who observed significant reductions in gray matter density and resting-state connectivity in the right dorsolateral prefrontal cortex (DLPFC), highlighting the interconnected nature of functional and structural changes induced by long-term heroin use.

Neuroimaging studies using advanced techniques have provided new insights into the cognitive and emotion processing impairments in OUD. Zhang et al. [27] used rs-fMRI along with support vector machine-based multi-voxel patterns to highlight significant differences in brain activation patterns involving the OFC, ACC, and amygdala in opioid users. Furthermore, graph theory analysis has shown how dysfunctional connectivity underlies the neural basis of behavioral deficits observed in opioid addiction. Liu et al. [28] identified several brain regions exhibiting dysfunctional connectivity in chronic opioid users during rs-fMRI, such as the prefrontal cortex, anterior cingulate cortex, supplementary motor area, ventral striatum, insula, amygdala, and hippocampus. Specifically, they found abnormal topological properties and dysfunctional connectivity within these regions, suggesting disruptions in the networks related to memory, inhibition, and motivation in the context of OUD.

## Identification of inconsistencies and methodological challenges

Using rs-fMRI to examine functional connectivity abnormalities in opioid addiction is an emerging method with the potential to inform novel treatment strategies. However, current research in this area presents inconsistencies and methodological challenges, including variable characteristics, small sample sizes, and a predominance of non-standardized analytical techniques, all of which can affect the reproducibility of findings.

One major limitation of prior studies using rs-fMRI in individuals with OUD is associated with the heterogeneity of participant samples, particularly regarding substance use characteristics such as the duration of opioid addiction, length of abstinence, history and number of relapses, or type of treatment (if any). For instance, studies such as Kuo et al. [29] and Xu et al. [30] examine heroin users under different treatment regimens (e.g., pharmacotherapy such as methadone maintenance treatment vs. medication-free therapeutic community) and different lengths of abstinence (short-term vs. long-term), respectively. The variability of these factors makes comparison between studies difficult, as different stages of addiction or recovery may impact functional connectivity in distinct ways. Kuo et al. [29] observed significant differences in the DMN between individuals undergoing MMT and those in a therapeutic community, highlighting the complex influence of treatment type. In contrast, Xu et al. [30] demonstrated that long-term abstinent heroin users exhibit partial recovery in functional connectivity of the midbrain, specifically the ventral tegmental area and substantia nigra. The study found that connectivity between these regions and areas associated with reward processing, such as the striatum and prefrontal cortex, showed signs of recovery with prolonged abstinence.

Another factor that contributes to inconsistencies in prior research findings is the variety of methodological approaches in fMRI. For example, Wang et al. [31] used seed-based connectivity analysis to explore the connectivity of the ventral anterior cingulate cortex in heroin users, while Yuan et al. [32] employed discrete cosine transform (DCT) to study network changes in abstinent heroin users. The preferences for specific analytical methods can lead to inconsistent findings. For example, seed-based analysis, which focuses on specific brain regions, may overlook interactions across global networks, while independent component analysis (ICA), which takes a more holistic approach, might miss region-specific data.

The scarcity of comprehensive longitudinal studies examining the long-term effects of heroin use and abstinence represents another significant gap in the literature. While many studies [31, 33] reveal brain connectivity differences between heroin users and controls, only one study to date has tracked connectivity changes over time [34]. The paucity of longitudinal data limits our understanding of the effects of long-term heroin use or abstinence on the deterioration or recovery of brain networks. Without sufficient data from longitudinal studies, it remains challenging to determine whether these alterations in brain activity are stable or progressive.

The lack of control of demographic factors in prior studies using rs-fMRI with opioid users also poses methodological challenges. Most studies fail to account for population-specific differences (e.g., age, sex, socioeconomic status, polydrug use etc.), which can influence brain structure and function. In addition, the lack of diverse participant pools in these studies hinders the generalizability of findings and limits a comprehensive understanding of the neurological underpinnings of opioid addiction.

Advancements in imaging technology, such as those discussed by Chai and Zhang [35], introduce new challenges and opportunities. Ultra-high-field MRI scanners and laminar fMRI offer intricate insights into brain micro-architecture but also present issues like signal loss and challenges regarding complex layer-specific analysis. Integrating such technologies into addiction research could propel the field forward but requires meticulous methodological enhancements and standardization.

Addressing these methodological challenges and inconsistencies would be pivotal for advancing our understanding of functional connectivity disruptions in opioid addiction. A concerted effort towards standardized methodologies, longitudinal studies, integrative findings, and diverse participant representation will pave the way for more robust research data, essential for developing effective addiction treatments. Large-scale collaborative studies such as ENIGMA [36], IMAGEN [37], and ABCD [38] have emerged as key initiatives addressing these challenges by integrating data from multiple sources and harmonizing methodologies.

## Aims

The comprehensive investigation of rs-fMRI in OUD focuses on examining how chronic opioid use alters functional connectivity within and between key brain networks. In line with this objective, the current study seeks to identify specific neural disruptions in the DMN, SN, and ECN in opioid users, which may guide the development of targeted interventions for OUD.

Despite growing evidence of resting-state functional connectivity (rs-FC) alterations in OUD, most existing studies lack well-controlled designs, limiting the ability to draw definitive conclusions.

To address this gap, our study employs a controlled experimental design with strict inclusion criteria, ensuring a well-matched sample of opioid users and healthy controls. This study is part of a larger ongoing international research project aimed at identifying common and substance-specific personality, neurobehavioral, neuroimaging, polygenic, and computational markers associated with opioid and stimulant use disorders [23]. For this report, we focus solely on functional connectivity in rs-fMRI in heroin users and non-substance-dependent controls.

## Materials and methods

### Participants

All participants were recruited and tested in Bulgaria between 2022 and 2024. Participants were recruited via information flyers distributed at substance abuse treatment facilities and social venues, personal referrals, and social media. Candidates interested in participating in the study were initially screened for eligibility over the phone. Exclusion criteria included less than 8 years of education, inability to read and write in Bulgarian, IQ < 75 [39], central nervous system illness or injury, head trauma with loss of consciousness of more than 30 min, open head injury of any type, presence of psychotic or mood disorders, current use of psychotropic medication, HIV-seropositive status, positive urine toxicology screen for amphetamines, methamphetamines, cocaine, opiates, methadone, cannabis, benzodiazepines, barbiturates, and MDMA, positive Breathalyzer test for alcohol, or any contraindications to MR scanning (e.g., metal fragments or implants, claustrophobia, pregnancy).

The sample consisted of 19 right-handed opioid users (OU) and 23 right-handed gender- and age-matched healthy controls (HC). All OU in the current sample met the criteria for lifetime OUD based on the Diagnostic and Statistical Manual of Mental Disorders [40] but had no history of any other substance use disorder except cannabis use disorder (if criteria were met at least 5 years prior to testing). In addition, all OU were abstinent at the time of testing: 12 (60%) were in short-term abstinence [1–3 months of abstinence] and 7 (40%) were in protracted abstinence [> 12 months of abstinence]. None of the participants who met the criteria for lifetime OUD were on opioid maintenance therapy at the time of testing. HC had no history of mental health disorders including substance use disorders according to the DSM-5.

### Procedures

The study was approved by the Institutional Review Boards of Virginia Commonwealth University, the Medical University in Sofia, and the Medical University of Plovdiv. Written informed consent was obtained from each participant. Abstinence from alcohol and drugs at the time of testing was confirmed using a breathalyzer test (Alcoscan AL7000) and urine toxicology screen for various substances, including amphetamines, barbiturates, benzodiazepines, cannabis, cocaine, MDMA, methadone, methamphetamines, and opiates. All participants were HIV-seronegative, determined by rapid HIV testing.

## MRI data acquisitions

The scanning procedure was implemented on a 3 T MRI system (GE Discovery 750w, General Electric, Boston, MA, USA). First, a high-resolution structural scan (Sag 3D T1 FSPGR) was performed with the following parameters: slice thickness = 1 mm, matrix size = 256 × 256, TE (echo time) = 2.21 ms, TR (repetition time) = 6.1 ms, and flip angle = 12°.

Second, a resting-state functional scan using a 2D Gradient echo sequence was obtained, with a slice thickness of 3 mm, matrix size = 64 × 64, TE = 30 ms, TR = 2000 ms, 36 slices, 192 volumes, and flip angle = 90°. Five dummy scans were acquired before each functional scan and discarded. During the functional series, all subjects were instructed to remain with their eyes closed and to not think of anything in particular.

## MRI data analysis

The processing of the structural and functional data was implemented using the CONN Toolbox [41] (See Supplement) running on the MATLAB platform for Mac. The processing steps are described in Supplement 1.

Seed selection for the between-group analysis was based on a combination of theoretical grounding from current literature and statistical validation from our own data. The selected seeds—Posterior Cingulate Cortex (PCC), Supramarginal Gyrus (SMG), and Superior Sensorimotor Cortex have been documented in previous neuroimaging studies as hubs disrupted in opioid use disorder (OUD).

## Results

### Descriptive statistics and group differences in demographic and opioid use variables (Table 1)

### Functional connectivity alterations of posterior cingulate cortex in the default mode network

**Fig. 1:**
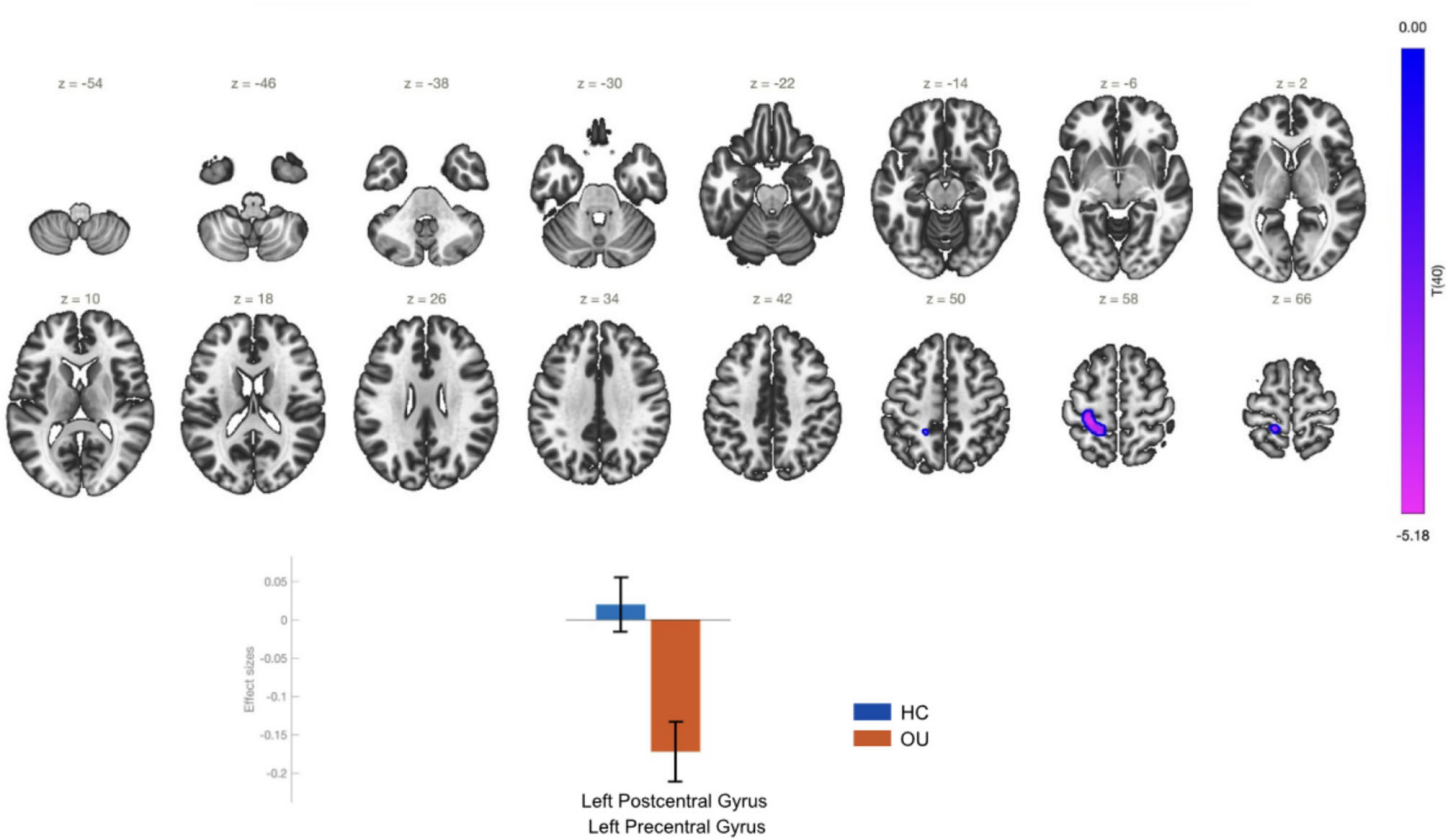
Effect sizes and functional connectivity of the PCC seed in the comparison OU > HC

The comparison between opioid users and the control group yielded a significant decrease in resting-state functional connectivity (rs-FC) between the PCC and the left pre- and post-central gyri in the OU group (Table 2; Fig. 1). The substantial negative effect size in the PCC suggests suppression of DMN activity. The PCC is a critical DMN hub implicated in internally-oriented cognition, self-referential thought and consciousness. Suppression here may reflect impaired introspection, emotion regulation, or metacognitive awareness commonly associated with addiction. The control group shows typical DMN activity, as evidenced by the slightly positive effect size.

**Table 1:** Demographic characteristics of the participants

	Healthy controls (*n* = 23)	Opioid users (*n* = 19)	Significance
Age (mean ± SD)	37.5 ± 5.5	37 ± 6	0.117^a^
Gender (M/F)	13/10	11/8	0.930^b^
Years of education	16.1 ± 2.6	12.6 ± 2.5	0.076^a^

*SD* standard deviation, ^a^*χ*^2^-test, ^*b*^Independent samples Mann–Whitney *U* test, *p < 0.05

**Table 2:** Overview of seed-to-voxel analysis of functional connectivity in OU

Seed	Regions within the clusters showing altered FC	Cluster-size (voxels)	Cluster-threshold (*p* < 0.05, FWE)	MNI coordinates *x*, *y*, *z*
Posterior cingulate cortex	Left postcentral gyrus, left precentral gyrus	268	0.00059	− 28 − 32 + 58
Superior marginal gyrus	Left Postcentral Gyrus, left superior parietal lobule	243	0.001493	− 42 − 40 + 58
	Brain stem	150	0.010458	+ 04 − 24 − 36
	Left temporal pole, left temporal fusiform cortex	99	0.037543	− 22 + 06 − 38
Superior sensory-motor cortex	Precuneus, Cingulate gyrus, vermis, lingual gyrus	701	0.0	− 02 − 50 + 06

### Functional connectivity of the superior sensory-motor cortex seed and DMN

**Fig. 2:**
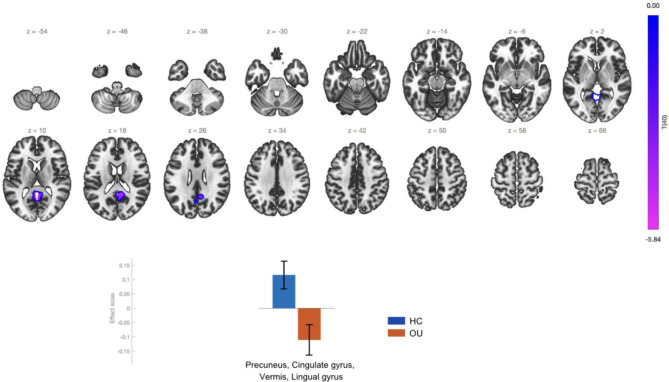
Effect sizes and functional connectivity of the superior sensory-motor cortex seed in the comparison OU > HC

There was a statistically significant reduction of the rs-FC between the Superior Sensory-Motor Cortex and Precuneus, Cingulate gyrus, Vermis, and Lingual gyrus in OU as opposed to HC (Table 1; Fig. 2). These alterations may reflect deficits in motor control, somatosensory processing, or broader network dysfunction. The aberrant connectivity with regions such as the precuneus and posterior cingulate gyrus implicates the role of DMN in the pathophysiology of opioid addiction.

### Functional connectivity of the supramarginal gyrus seed in the salience network

**Fig. 3:**
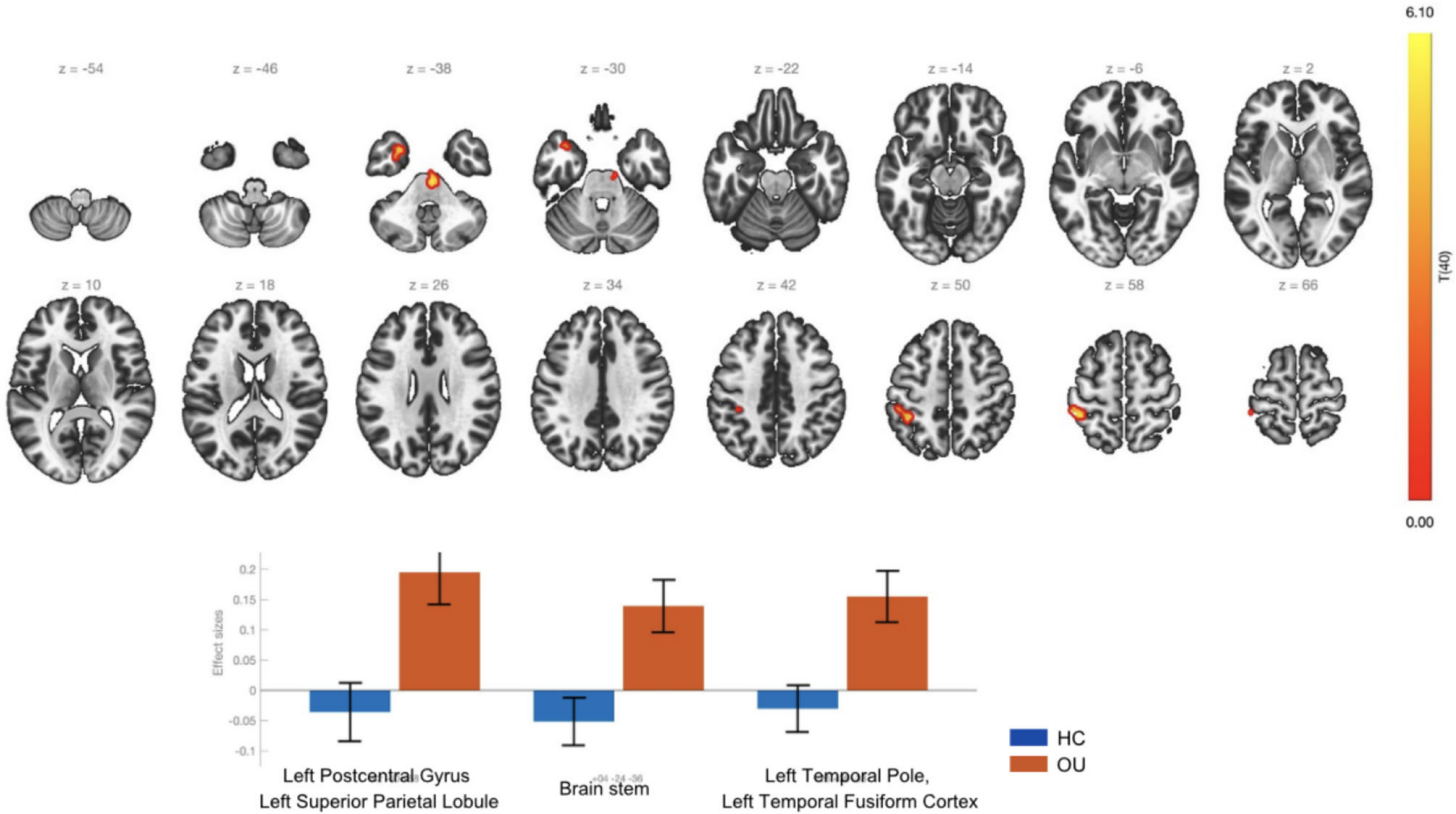
Effect sizes and functional connectivity supramarginal gyrus seed in the comparison OU > HC

The results demonstrated a statistically significant increase in rs-FC between the SMG and the left Postcentral Gyrus, left Superior Parietal Lobule, Brain stem, left Temporal Pole, and left Temporal Fusiform Cortex in the OU group in comparison to the control group. This may reflect enhanced recruitment of the salience network in substance use disorders, where individuals assign exaggerated importance to internal or external stimuli (e.g., drug-related cues or cravings) (Fig. 3).

## Discussion

Our findings of altered functional connectivity (FC) in the default mode network (DMN), salience network (SN), and sensory-motor networks among opioid users align with a growing body of literature that underscores the pervasive neural disruptions associated with OUD. For instance, Ma et al. [42] similarly reported aberrant DMN connectivity in heroin-dependent individuals, with notable suppression of the posterior cingulate cortex (PCC), consistent with our observations. This convergence suggests that PCC hypoactivity may serve as a potential neural marker of impaired introspection and emotion regulation in OUD, which might contribute to the persistent cognitive deficits documented in chronic opioid users [24, 43]. However, while Ma et al. focused primarily on structural and functional connectivity within the DMN, our study extends these findings by demonstrating disruptions across multiple interacting networks, including the SN and sensory-motor regions, which may reflect a broader dysregulation in salience attribution and sensory integration.

Comparatively, Liu et al. [13] explored dysfunctional connectivity in heroin-dependent individuals and found that heroin cues exacerbated abnormal responses in brain regions tied to reward processing and attention, such as the insula and prefrontal cortex. This aligns with our observation of an overactive SN in opioid users, who may prioritize drug-related stimuli over natural rewards, as evidenced by altered resting-state FC (rs-FC) in the Superior Parietal Lobule and Supramarginal Gyrus. Unlike Liu et al., who emphasized cue-induced responses, our resting-state approach suggests that these salience network abnormalities persist even in the absence of explicit drug cues, pointing to a more enduring neurobiological vulnerability in OUD.

Additionally, our results resonate with the longitudinal findings from Zhang et al. [34], who documented partial recovery of FC between the dorsolateral prefrontal cortex (DLPFC) and insula in abstinent heroin users, alongside reduced craving. While our cross-sectional design limits inferences about recovery trajectories, the suppression of DMN activity and heightened SN engagement in our sample of active opioid users could represent an earlier stage along this continuum, prior to abstinence-driven neuroplasticity. This comparison highlights the potential for rs-fMRI to track dynamic changes in network connectivity over time, a direction we aim to pursue in future longitudinal analyses.

In contrast, some studies report findings that diverge from ours in scope or specificity. For example, Yuan et al. [26] identified gray matter deficits alongside resting-state abnormalities in abstinent heroin users, suggesting that structural changes may compound the functional disruptions we observed. Our study, however, did not assess structural integrity, which could account for differences in the magnitude of network-level effects. Integrating multimodal imaging, as advocated by Koob and Volkow [2] and Canario et al. [11], could clarify how structural and functional alterations interact in OUD, offering a more comprehensive view of addiction pathophysiology.

Furthermore, our finding of sensory-motor network disruptions aligns with Pandria et al. [25], who noted resting-state abnormalities in heroin-dependent individuals extending beyond cognitive and reward circuits to sensory processing regions. This overlap suggests that OUD impacts a wide array of neural systems, potentially contributing to the sensory distortions and motor impairments observed clinically in chronic users. However, where Pandria et al. broadly surveyed resting-state changes, our study pinpoints specific hubs like the Supramarginal Gyrus, suggesting targeted loci for future therapeutic interventions such as neurofeedback or transcranial magnetic stimulation.

These comparisons with prior work underscore the consistency of DMN and SN involvement across OUD studies, while highlighting our contribution: a detailed mapping of rs-FC disruptions across sensory, cognitive, and emotional domains. By identifying these network-specific changes, our findings support the Addictions Neuroclinical Assessment (ANA) framework proposed by Kwako et al. [3], which seeks to define addiction through neurobiological domains rather than by solely symptom-based criteria. Integrating our rs-fMRI data with phenotypic and genotypic data as planned in our ongoing study, could further align with efforts to delineate addiction biotypes [44], akin to those proposed for depression and anxiety by Williams [45]. Such approaches may bridge the gap between the generalized network disruptions observed here and the heterogeneous presentations of OUD in clinical settings.

To fully realize the translational potential of these findings, future research should address methodological variations across studies—such as differences in abstinence duration, drug exposure history, and imaging protocols—as highlighted by Temtam et al. [46]. Standardizing these factors, alongside expanding sample diversity and longitudinal follow-up, will refine our understanding of how opioid addiction reshapes brain connectivity and inform the development of personalized interventions targeting these disrupted networks.

## Limitations

There is a several limitations that need to be acknowledged. The sample size in our study w as relatively small, which limits the inference power and generalizability. However, we regard the reported data as preliminary findings fostering the critical arguments in our literature review. Another caveat of this study is that the group of patients with OUD consists of individuals in both long- and short-term remission. Data collection is continuing and we plan to explore the effects of length of abstinence once we are sufficiently powered to do so.

## Conclusion

The article underscores the significant impact of opioid addiction on brain connectivity, particularly within the default mode network (DMN), salience network (SN), and sensory-motor network (SMN). Prior studies highlight the interplay between these networks and addiction-related behaviors, with disrupted connectivity manifesting as both functional and structural changes in the brain.

Building upon previous findings, our ongoing fMRI study, not only corroborates previous research but also offers new insights into the specific disruptions observed in abstinent individuals with OUD. Our analysis reveals altered functional connectivity among hubs of the SN, the DMN, and the sensorimotor network which may reflect of deficits in sensory-motor integration that are crucial for adaptive behavior and successful recovery. The findings indicating SMN involvement were unexpected and need to be investigated further.

## Supplementary Information

Below is the link to the electronic supplementary material.

Supplementary file1 (DOCX 35 KB)

## Data Availability

The data presented in this study are available on request from the corresponding author.
