# Supplementary material for: Functional connectivity in resting-state fMRI (rs-fMRI) in opioid use disorder
Source: Eur Phys J Spec Top. 2025 Mar 25;234(15):4127–37. doi: 10.1140/epjs/s11734-025-01591-2 (PMC12518422; doi:10.1140/epjs/s11734-025-01591-2)
Supplement: Supplementary file 1 — Supplementary file1 (DOCX 35 KB) [file 11734_2025_1591_MOESM1_ESM.docx]

**SUPPLEMENT**

**MRI data analysis:**

Analyses of fMRI data were performed using CONN [1] (RRID:SCR_009550) release 22.v2407 [2] and SPM [3] (RRID:SCR_007037) release 12.dev.

**4.1 Preprocessing**: Anatomical and resting-state data were preprocessed using the default preprocessing pipeline [4] including realign and unwarp, slice timing correction, outlier detection (artifact-detection-tool-based), direct segmentation and MNI-space normalization,  and smoothing using 6 mm FWHM Gaussian kernel.

Functional data were realigned using SPM realign & unwarp procedure [5], where all scans were coregistered to a reference image (first scan of the first session) using a least squares approach and a 6 parameter (rigid body) transformation [6], and resampled using b-spline interpolation to correct for motion and magnetic susceptibility interactions. Temporal misalignment between different slices of the functional data (acquired in ascending order) was corrected following SPM slice-timing correction (STC) procedure [7,8], using sinc temporal interpolation to resample each slice BOLD timeseries to a common mid-acquisition time. Potential outlier scans were identified using ART [9] as acquisitions with framewise displacement above 0.9 mm or global BOLD signal changes above 5 standard deviations [10,11], and a reference BOLD image was computed for each subject by averaging all scans excluding outliers. Functional and anatomical data were normalized into standard MNI space, segmented into grey matter, white matter, and CSF tissue classes, and resampled to 2 mm isotropic voxels following a direct normalization procedure [11,12] using SPM unified segmentation and normalization algorithm [13,14] with the default IXI-549 tissue probability map template. Last, functional data were smoothed using spatial convolution with a Gaussian kernel of 8 mm full width half maximum (FWHM).

**4.2. Denoising**: In addition, functional data were denoised using the standard denoising pipeline [15] including the regression of potential confounding effects characterized by white matter timeseries (5 CompCor noise components), CSF timeseries (5 CompCor noise components), motion parameters and their first order derivatives (12 factors)[16] outlier scans (below 26 factors)[10], session effects and their first order derivatives (2 factors), and linear trends (2 factors) within each functional run, followed by bandpass frequency filtering of the BOLD timeseries[17] between 0.008 Hz and 0.09 Hz.

**4.3. First-level analysis**: For each subject seed-based connectivity maps (SBC) and ROI-to-ROI connectivity matrices (RRC) were estimated characterizing the patterns of functional connectivity with 164 HPC-ICA networks [2] and Harvard-Oxford atlas ROIs [20]. Functional connectivity strength was represented by Fisher-transformed bivariate correlation coefficients from a weighted general linear model (weighted-GLM [21]), defined separately for each pair of seed and target areas, modeling the association between their BOLD signal timeseries. In order to compensate for possible transient magnetization effects at the beginning of each run, individual scans were weighted by a step function convolved with an SPM canonical hemodynamic response function and rectified.

The output data of the steps were manually reviewed for each of the forty-two participants.

**4.4. Group-level analyses** were performed using General Linear Model (GLM[22]). The between-group parametric statistics were random field theory-based with a voxel threshold p < 0.001 (uncorrected) and a cluster threshold p < 0.05 (cluster-size Family-Wise Error (FWE) corrected). Voxel-level hypotheses were evaluated using multivariate parametric statistics with random effects across subjects and sample covariance estimation across multiple measurements. Inferences were performed at the level of individual clusters (groups of contiguous voxels). Cluster-level inferences were based on parametric statistics from Gaussian Random Field theory [23,24]. Results were thresholded using a combination of a voxel threshold p < 0.001 and a cluster threshold p-FWE (Family-Wise Error corrected) < 0.05 [25]. In addition, effect values representing the connectivity average difference for each seed and target cluster were computed using the default settings in the CONN Toolbox (Table 1, T-values).

Table 1: Between-group differences of the selected seeds

| **Seed** | **Regions within the clusters showing altered FC** | **Cluster-size**  **(voxels)** | **Cluster-threshold (p<0.05, FWE)** | **MNI coordinates x,y,z** | **T-values** |
| --- | --- | --- | --- | --- | --- |
| Posterior Cingulate Cortex | Left Postcentral Gyrus, Left Precentral Gyrus | 268 | 0.00059 | -28 -32 +58 | -6.13 |
| Superior Marginal Gyrus | Left Postcentral Gyrus, Left Superior Parietal Lobule | 243 | 0.001493 | -42 -40 +58 | 5.43 |
|  | Brain stem | 150 | 0.010458 | +04 -24 -36 | 5.49 |
|  | Left Temporal Pole, Left Temporal Fusiform Cortex | 99 | 0.037543 | -22 +06 -38 | 5.44 |
| Superior Sensory-motor Cortex | Precuneus, Cingulate gyrus, Vermis, Lingual gyrus | 701 | 0.0 | -02 -50 +06 | -5.32 |

**References:**

[1] Whitfield-Gabrieli, S., & Nieto-Castanon, A. (2012). Conn: a functional connectivity toolbox for correlated and anticorrelated brain networks. Brain connectivity, 2(3), 125-141.

[2] Nieto-Castanon, A. & Whitfield-Gabrieli, S. (2022). CONN functional connectivity toolbox: RRID SCR_009550, release 22. doi:10.56441/hilbertpress.2246.5840.

[3] Penny, W. D., Friston, K. J., Ashburner, J. T., Kiebel, S. J., & Nichols, T. E. (Eds.). (2011). Statistical parametric mapping: the analysis of functional brain images. Elsevier.

[4] Nieto-Castanon, A. (2020). FMRI minimal preprocessing pipeline. In Handbook of functional connectivity Magnetic Resonance Imaging methods in CONN (pp. 3–16). Hilbert Press.

[5] Andersson, J. L., Hutton, C., Ashburner, J., Turner, R., & Friston, K. J. (2001). Modeling geometric deformations in EPI time series. Neuroimage, 13(5), 903-919.

[6] Friston, K. J., Ashburner, J., Frith, C. D., Poline, J. B., Heather, J. D., & Frackowiak, R. S. (1995). Spatial registration and normalization of images. Human brain mapping, 3(3), 165-189.

[7] Henson, R. N. A., Buechel, C., Josephs, O., & Friston, K. J. (1999). The slice-timing problem in event-related fMRI. NeuroImage, 9, 125.

[8] Sladky, R., Friston, K. J., Tröstl, J., Cunnington, R., Moser, E., & Windischberger, C. (2011). Slice-timing effects and their correction in functional MRI. Neuroimage, 58(2), 588-594.

[9] Whitfield-Gabrieli, S., Nieto-Castanon, A., & Ghosh, S. (2011). Artifact detection tools (ART). Cambridge, MA. Release Version, 7(19), 11.

[10] Power, J. D., Mitra, A., Laumann, T. O., Snyder, A. Z., Schlaggar, B. L., & Petersen, S. E. (2014). Methods to detect, characterize, and remove motion artifact in resting state fMRI. Neuroimage, 84, 320-341.

[11] Nieto-Castanon, A. (submitted). Preparing fMRI Data for Statistical Analysis. In M. Filippi (Ed.). fMRI techniques and protocols. Springer. doi:10.48550/arXiv.2210.13564

^[12]^ [12] Calhoun, V.D., Wager, T.D., Krishnan, A., Rosch, K.S., Seymour, K.E., Nebel, M.B., Mostofsky, S.H., Nyalakanai, P. and Kiehl, K. (2017). The impact of T1 versus EPI spatial normalization templates for fMRI data analyses (Vol. 38, No. 11, pp. 5331-5342).

[13] Ashburner, J., & Friston, K. J. (2005). Unified segmentation. Neuroimage, 26(3), 839-851.

[14] Ashburner, J. (2007). A fast diffeomorphic image registration algorithm. Neuroimage, 38(1), 95-113.

[15] Nieto-Castanon, A. (2020). FMRI denoising pipeline. In Handbook of functional connectivity Magnetic Resonance Imaging methods in CONN (pp. 17–25). Hilbert Press.

[16] Friston, K. J., Williams, S., Howard, R., Frackowiak, R. S., & Turner, R. (1996). Movement-related effects in fMRI time-series. Magnetic resonance in medicine, 35(3), 346-355.

[17] Hallquist, M. N., Hwang, K., & Luna, B. (2013). The nuisance of nuisance regression: spectral misspecification in a common approach to resting-state fMRI preprocessing reintroduces noise and obscures functional connectivity. Neuroimage, 82, 208-225.

[18] Behzadi, Y., Restom, K., Liau, J., & Liu, T. T. (2007). A component based noise correction method (CompCor) for BOLD and perfusion based fMRI. Neuroimage, 37(1), 90-101.

[19] Chai, X. J., Nieto-Castanon, A., Ongur, D., & Whitfield-Gabrieli, S. (2012). Anticorrelations in resting state networks without global signal regression. Neuroimage, 59(2), 1420-1428.

[20] Desikan R.S., Ségonne F., Fischl B., Quinn B.T., Dickerson B.C., Blacker D., Buckner R.L., Dale A.M., Maguire R.P., Hyman B.T., Albert M.S., & Killiany R.J. (2006) An automated labeling system for subdividing the human cerebral cortex on MRI scans into gyral based regions of interest. Neuroimage 31(3):968-980

[21] Nieto-Castanon, A. (2020). Functional Connectivity measures. In Handbook of functional connectivity Magnetic Resonance Imaging methods in CONN (pp. 26–62). Hilbert Press.

[22] Nieto-Castanon, A. (2020). General Linear Model. In Handbook of functional connectivity Magnetic Resonance Imaging methods in CONN (pp. 63–82). Hilbert Press.

[23] Worsley, K. J., Marrett, S., Neelin, P., Vandal, A. C., Friston, K. J., & Evans, A. C. (1996). A unified statistical approach for determining significant signals in images of cerebral activation. Human brain mapping, 4(1), 58-73.

[24] Nieto-Castanon, A. (2020). Cluster-level inferences. In Handbook of functional connectivity Magnetic Resonance Imaging methods in CONN (pp. 83–104). Hilbert Press.

[25] Chumbley, J., Worsley, K., Flandin, G., & Friston, K. (2010). Topological FDR for neuroimaging. Neuroimage, 49(4), 3057-3064.
